# A cusp catastrophe model for analyzing sudden channel shifting in wandering reach of the Lower Yellow River

**DOI:** 10.1038/s41598-023-44937-3

**Published:** 2023-10-16

**Authors:** Xiangping Zhang, Enhui Jiang, Junhua Li, Tingkui Zhang, Ruixun Lai

**Affiliations:** 1 Yellow River Institute of Hydraulic Research, YRCC, Zhengzhou, 450003 China; 2 Key Laboratory of Lower Yellow River Channel and Estuary Regulation, MWR, Zhengzhou, 450003 China; 3 College of Water Resources and Civil Engineering, Zhengzhou University, Zhengzhou, 450001 China

**Keywords:** Hydrology, Geomorphology

## Abstract

Sudden channel shifting of wandering rivers poses significant challenges for river engineering, flood control strategies, and the security of water resources. This study proposes a novel analytical model to quantitatively assess such channel shifts based on the cusp catastrophe theory. Utilizing bathymetric data from 93 river sections collected biannually between 2015 and 2019, a comprehensive investigation of channel instability in the wandering reach of the Lower Yellow River was conducted. The results show that both lateral and longitudinal stabilities in the Huayuankou-Gaocun section were generally favorable. For the most part of the selected sections demonstrated a propensity for gradual change. Out of the 93 sections, totally 45 were identified as being susceptible to sudden channel shifts. These vulnerable sections were predominantly located in two highly active reaches—Xiaodabin-Jinglonggong and Huayuankou-Zhaolanzhuang—during the period from 2015 to 2017, excluding the 2018–2019 non-flood season. In the latter non-flood period, nearly half of the sections in the downstream reach from Jinglonggong displayed instability. Our findings are consistent closely with empirical observations from the Lower Yellow River. Additionally, the changed flow and sediment conditions in the 2015–2019 and the efficiency of guide works have significant effects on the stability of river channels.

## Introduction

**Figure 1:**
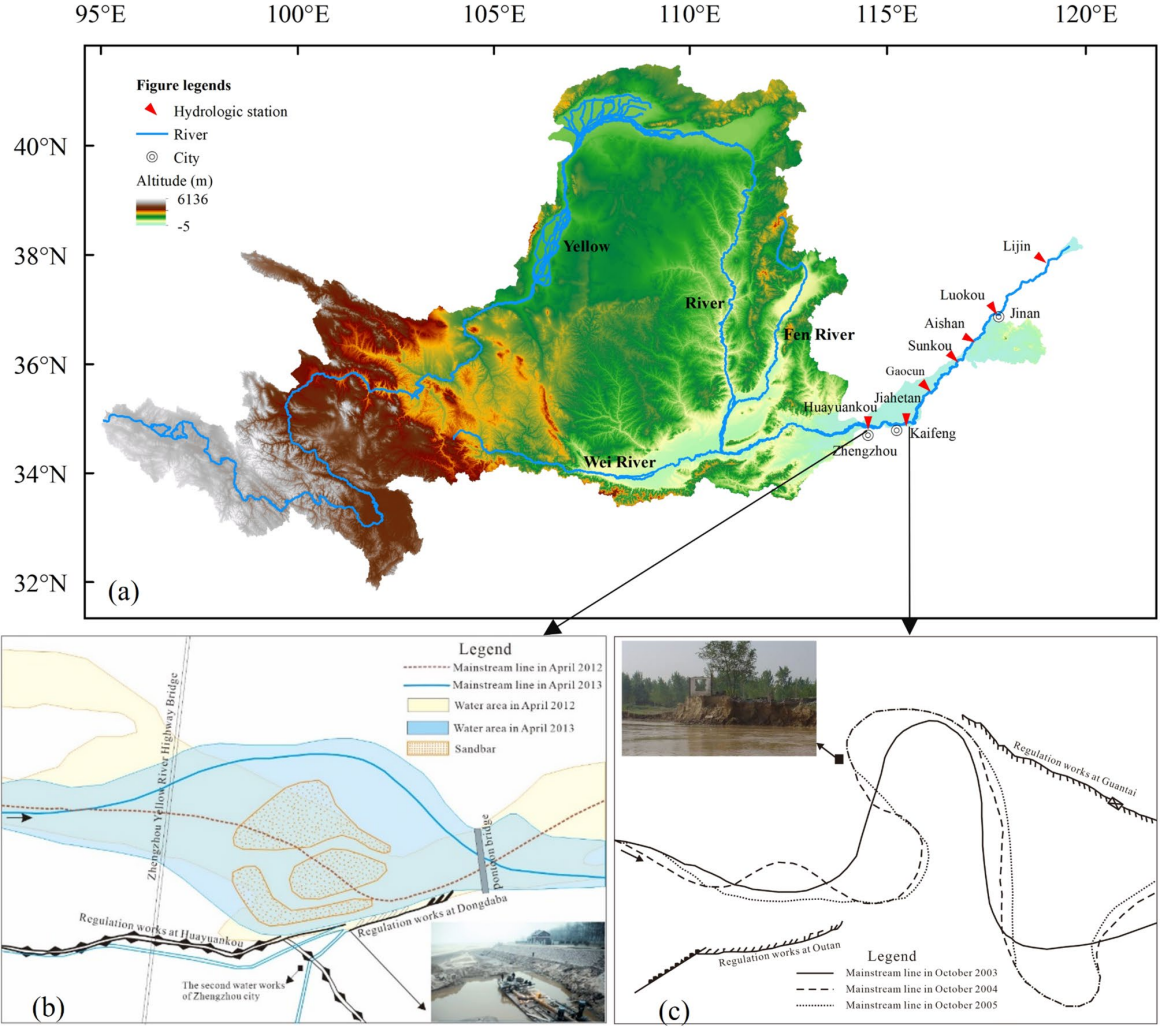
Sudden channel shifting of the lower Yellow River. The map was generated by the authors with the help of ArcGIS 10.8 (http://www.esri.com/software/arcgis) and does not require any permission from anywhere.

Sudden channel shifting represent a typical geomorphological phenomenon in the evolution of wandering river, posing significant risks to river regulation works, water supply systems, and riparian villages, especially in densely populated regions globally^1–4^. For instance, in 2018, a channel shifting in the Ganga River led to the disappearance of dozens of houses within a 72-h period in Gopalpur, India^1,5^. Similarly, in 2013, the channel of the Lower Yellow River (LYR) adjacent to Zhengzhou city was shifted 2 km to the north. Consequently, the original water intake point on the river's southern bank could not obtain enough water supply from the end of November 2013 to January 2014, resulting in water shortages for approximately 4.5 million Zhengzhou residents^2,6^, as illustrated in Fig. 1a,b. Additionally, sudden channel shifting can cause the emergence of malformed meanders of wandering river, severely threatening safety of riparian villages, as shown in Fig. 1a,c^7,8^. The map in Fig. 1 was prepared by the co-authors with the help of ArcGIS 10.8 (http://www.esri.com/software/arcgis).

River channel shifting indicates a positional change of the channel mainstream due to changed water flow and sediment load, boundary conditions and it is a key issue of global fluvial geomorphology research^9–17^. Most river channel shifting is gradual with mild or moderate channel migration rate and relatively stable river regime^18–20^. River channel shifting, especially in the case of wandering rivers, is highly complex. Sudden channel shifts in wandering rivers often occur when there is a high channel migration rate and an unstable river regime, particularly when certain contributing factors reach a critical threshold. Due to variations in channel migration distance and rate across different sections of wandering rivers, establishing a standardized criterion for the identifying and quantifying sudden channel shifting is a challenging task.

River channel shifting has been extensively investigated and studied by researchers utilizing various metrics for characterization. Typical parameters include channel migration distance and channel migration rate of the river mainstream or thalwegs usually were used to describe river channel shifting^21–25^. Additional indices, such as channel sinuosity index^16,17^, bifurcation angles^26^, and lateral migration rate^27^ were developed to describe the channel changes in river morphology. For instance, Kong developed anabranching indices, braid-channel ratio, sinuosity, and lateral migration rate to investigate marked morphological changes in the LYR^27^. Liu introduced a novel quantitative metric called the relative stability index of river regimes to assess the stability of river channels^23^. Sarif focused on the meandering of the river sinuosity and erosion–deposition dynamics to river channel migration^1^. Raj evaluated the longitudinal changes and the variation of the sinuosity index of River Ganga from Bhagalpur to Kahalgaon during the period of 1973–2018^16^. Despite extensive studies, there is still inconsistent understanding regarding the types, migration rates, and contributory factors involved in river channel shifting, especially in wandering rivers. While previous research has documented patterns of sudden channel shifting that have led to the loss of villages or cities and levee failures, a limitation of their findings is that they have not identified other potential instances of sudden channel shifting or elucidated their underlying mechanisms^3,6^.

To date, research on sudden channel shifting presents substantial challenges due to the phenomena’s abrupt onset, rapid evolution, and inherent variability. These factors contribute to difficulties in both measurement and quantitative description, particularly given the uncertainties of the timing and processes of such shifts. The previous study on channel shifting focused on the gradual process of channel migration^28–33^. In recent years, with the development of nonlinear science, the catastrophic aspects of the riverbed evolution have attracted increasing attention. Catastrophe theory has been deployed to investigate various catastrophic phenomena with still unknown internal mechanisms. Thornes utilized catastrophe theory to qualitatively assess the abrupt changes in rivers in Spain's arid regions^34^. Richards applied the same theory to explore the river regime formation^35^. Graf used a cusp catastrophe model to describe transformations among straight, meandering, and wandering river patterns and proposed that both sudden and gradual changes exist in the river pattern transformation^36,37^. Cui established a cusp catastrophe model for debris flow initiation, suggesting multiple pathways for the onset of quasi-debris flows^38^. Wang developed a similar model of sediment initiation to explain mechanisms of different sediment initiation, taking the flow motion intensity parameters as state variables^39^. Yang took the Shields number as a state variable; the sand Reynolds number was set to − 0.3, and the dimensionless parameter m_dm_/d_0_ (m is the density coefficient, *dm* is the average particle size) as a two-control variable and established the cusp catastrophe model to reflect the law of non-uniform sediment initiation^40^. Song presented a grey cusp catastrophe model to analyze the stability of the fluvial process in the Qiangtangjiang estuary, with the longitudinal stability index of the estuary characterized by the river bed degradation and aggradation intensity and the transversal stability index by the ratio of the river width to the river depth^41,42^. Xiao reported a cusp catastrophe model for predicting alluvial channel stability^43^. Based on the characteristics of sediment movement with the submarine pipeline scour state, a nonlinear relation formula between the Shields number, the non-dimensional number, and the scour depth under steady flow was proposed using the cusp catastrophe theory^44^. Based on difficulties in the study of sudden channel shifting and advantages of catastrophe theory, the channel sudden shifting model based on the cusp catastrophe theory is developed in this research after the characteristics of sudden channel shifting of the wandering river were analyzed. This approach has been applied to the wandering reach of the LYR in China.

The aims of this study are: (i) to develop a new methodology for analyzing sudden channel shifting of the wandering river; (ii) to analyze the internal mechanism of the sudden channel shifting of the wandering reach of the LYR; (iii) to discuss the consistence of the sudden channel shifting of the wandering reach of the LYR over the period 2015–2019 with field observations and multi-sources remote sensing images.

## Materials and methods

### Study area

The Yellow River, with a total length of 5464 km, ranks as the second-longest river in China^45^. It is notable for its significant sediment load in the world, with a long-term average annual sediment load of 1.6 billion tons^46^. The LYR, from Mengjin to Lijin is divided into three morphologically distinct sub-reaches, respectively the wandering, transitional and meandering reaches^47,48^.

**Figure 2:**
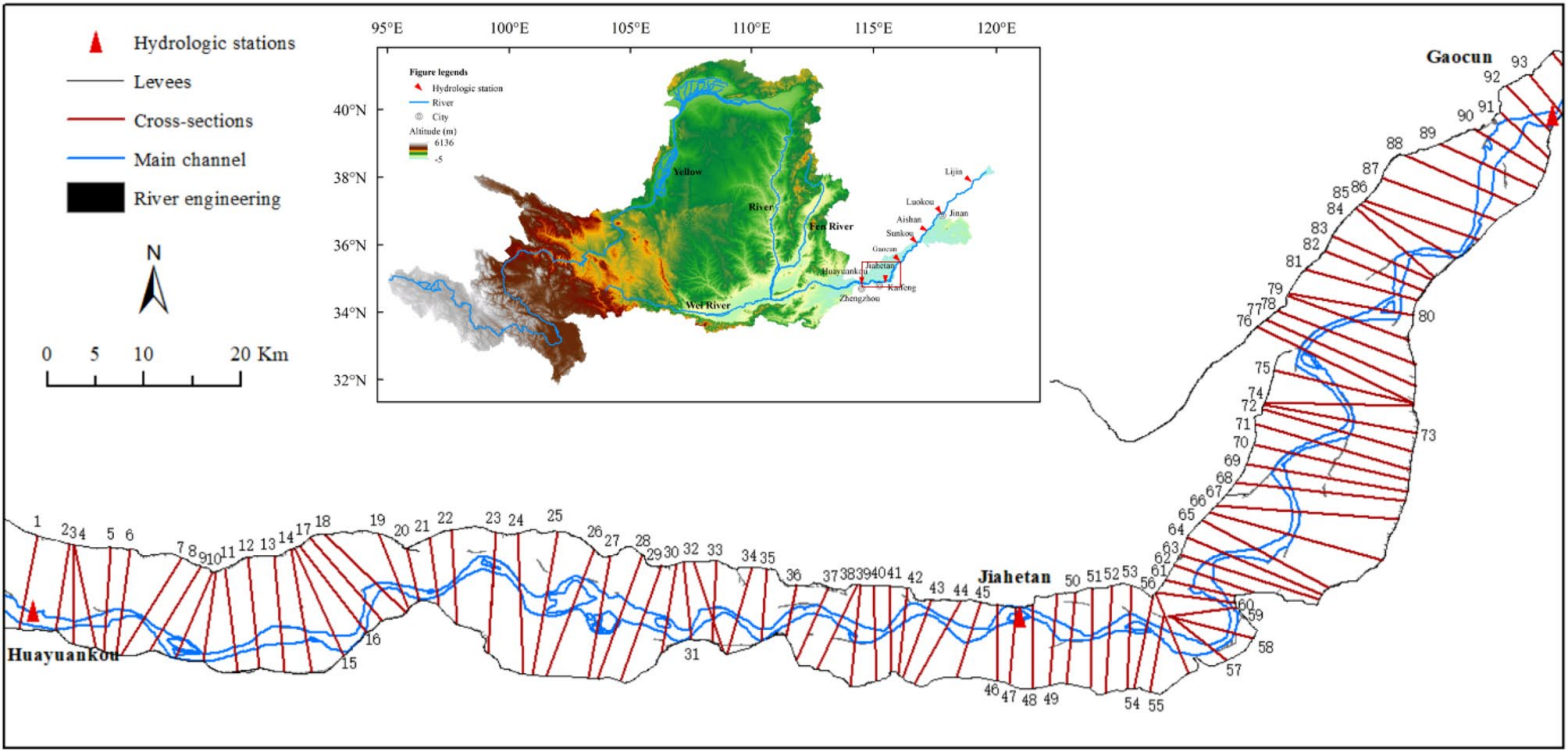
Study area and cross-sectional settings. 1. Huayuankou, 2. Pochezhuang, 3. Zhaolanzhuang, 4. Qibao, 5. Shuangkai, 6. Babao, 7. Shenzhuang, 8. Shiqiao, 9. Madu, 10. Laitongzhai, 11. Sanba, 12. Yangqiao, 13. Sunzhuang, 14. Wuzhuang, 15. Huanglianji, 16. Zhaokou, 17. Liubao, 18. Baochang, 19. Maoan, 20. Xinzhai, 21. Yueshi, 22. Xiaodabin, 23. Sanguanmiao, 24. Heishi, 25. Guozhuang, 26. Doumen, 27. Xuzhuang, 28. Weicheng, 29. Xisanli, 30. Huihuizhai, 31. Yudian, 32. Heigangkou, 33. Niezhuang, 34. Jinglonggong, 35. Gaozhuzhuang, 36. Liuyuankou, 37. Dingzhuang, 38. Fanzhuang, 39. Wangan, 40. Sizhuang, 41. Peilou, 42. Gucheng, 43. Chenqiao, 44. Yuanfang, 45. Fujunsi, 46. Caogang, 47. Changmenkou, 48. Xiaohetou, 49. Qingheji, 50. Diwanzha, 51. Zhuzhai, 52. Kuzhuang, 53. Guantai, 54. Jiahetan, 55. Ewan, 56. Sanyizhai, 57. Dinggedang, 58. Nanbeizhuang, 59. Dongbatou, 60. Xinzhuang, 61. Yangzhuang, 62. Leiji, 63. Chanfang, 64. Dianji, 65. Xizhangji, 66. Zuozhaizha, 67. Limenhzuang, 68. Youfangzhai, 69. Machang, 70. Jinggang, 71. Dawangzhai, 72. Wanggaozhai, 73. Xindian, 74. Liuheji, 75. Dongheigang, 76. Mazhai, 77. Changxingji, 78. Zhulin, 79. Shitouzhuang, 80. Xiezhaizha, 81. Xiaosuzhuang, 82. Yangxiaozhai, 83. Yulin, 84. Huangzhai, 85. Wuqiu, 86. Xibaocheng, 87. Zhaodi, 88. Zhangzhai, 89. Hedao, 90. Shuangjing, 91. Qingzhuang, 92. Shiziyuan, 93. Gaocun.

This study specifically focuses on the wandering reach of the LYR between Huayuankou and Gaocun stations covering a span of 174 km (Fig. 2). Characterized by a highly unstable river regime, the macrochannel width of this reach varies between 5 and 24 km, and features an array of sandbars and islands in the river course^49^. Although the reach is already well-developed by the upstream Xiaolangdi reservoir, levees on both river banks, and many flow guide works along the river course, the mainstream is still shifting. The channel morphology frequently changes in response to varying degrees of water–sediment conditions. Classified as a sand-bed and fine-sand suspended-load river, the average river slope of this section is approximately 0.20 m km^−1^.

To conduct river geometrical and bathymetry measurements, cross-sections along the channel with approximately equal intervals and crosslines perpendicular to the main flow direction were set in the LYR^50^. Figure 2 drawn by using ArcGIS (version 10.8) illustrates 93 cross-sections fixed up the wandering reach of LYR. The high-flow period typically spans from May to September, whereas the low-flow period extends from October to April of the subsequent year. Usually, the geometrical and bathymetry measurements of the river were routinely implemented by the Yellow River Conservancy Commission (YRCC) twice per year, the first in April or May prior to the high-flow period and the second in October or November following the high-flow period.

### Methods

**Figure 3:**
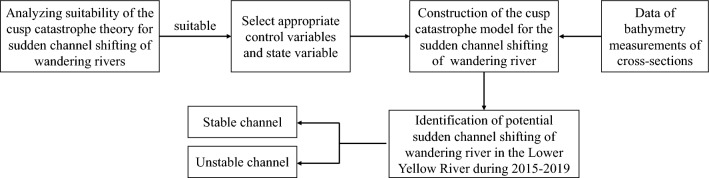
Flowchart of the methodology.

Figure 3 illustrates the methodological framework for analyzing sudden channel shifting in wandering rivers. Initially, we assessed the suitability of cusp catastrophe theory for explaining sudden channel shifts in wandering rivers. Subsequently, we identified the relevant control and state variables. We constructed the cusp catastrophe model to describe sudden channel shifting in wandering rivers. Finally, we identified instances of potential sudden channel shifting in the wandering river of the LYR during the period from 2015 to 2019.

#### Characteristics of the sudden channel shifting for wandering rivers

##### Mutability

Sudden channel shifting in rivers represent a localized, catastrophic change process in river dynamics^9^. The channel migration of a wandering river can be considered as a system developed toward a dynamic equilibrium. The system has two states of change, including gradual change and sudden change namely. The system change is governed primarily by two factors, including channel erosion and sedimentation, and the cross-section shape is affected by water and sand. Under these two factors, the system state usually changes gradually. However, if one of them exceeds certain critical thresholds, a sudden change of the system state occurs. Mutation is a shortcut to push forward the rapid development of the system.

##### Dimorphism

The channel migration dynamics of a meandering river can achieve a state of equilibrium through the interplay of sediment deposition and erosion. This system exhibits dimorphism in fluvial bedform evolution. When the sediment load in the water flow exceed the river's carrying capacity, deposition occurs on the riverbed. However, the sedimentation is not unbounded, and is self-regulates toward an equilibrium state. Similarly, from an erosional perspective, scouring processes are also self-limiting and is not indefinite.

##### (3) Hysteresis

In the context of channel migration dynamics of wandering river, sudden channel shifting is a shortcut of river evolution. Mutation necessitates a higher energy input for reversal, rendering a return along the original trajectory unfeasible. This gives rise to a hysteresis and irreversible phenomenon within the system. Specifically, the critical value that triggers a system transition in the forward direction is insufficient to revert the system to its original state during the reverse process. Consequently, the reverse operation lags temporally behind the forward operation, meaning that the system's state change exhibits a delay relative to changes in the control variables. Once a transition occurs, reversion to the prior state becomes hard.

##### (4) Divergence

In physical systems, a phenomenon known as divergence describes the instability that arises in the path variation of parameters. Within the framework of a wandering river's channel migration dynamics, the system remains gradually changing as long as the value of channel erosion–deposition and the cross-section shape vary modestly away from a critical value. However, when this critical threshold is reached or exceeded, minor alterations in channel erosion–deposition, or cross-sectional morphology can induce significant shifts in the channel migration state, potentially leading to drastic changes in both its strength and structural integrity. In essence, minor fluctuations in control variables can precipitate either incremental or abrupt changes in state variables, thereby destabilizing the system. Sudden changes lead to the accelerated effect of channel shifting under the influence of system divergence.

##### Multiple paths

In the channel migration dynamics of a wandering river, multiple paths of moving forward exist from one system state to another. Mutations occur within a specific region, which we term the “mutation region”. In contrast, the system undergoes only incremental changes in state or gradually change outside the “mutation region”, encompassing both critical and non-critical regions.

**Figure 4:**
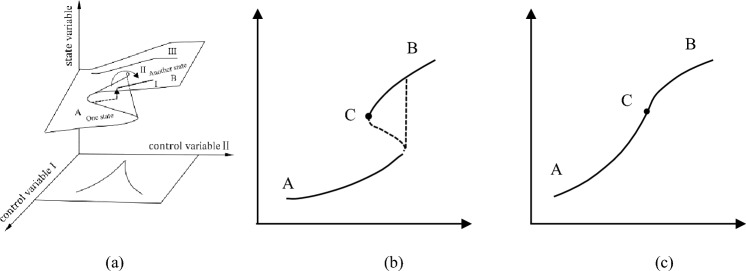
Multiple paths of channel shifting.

To illustrate this, taking the cusp catastrophe model as an example, if A represents an initial state of channel shifting, B represents another state following sudden channel shifting, and an intermediate state ‘C’ exists, as shown in Fig. 4. Transitioning from state A to B via C is considered as a ‘forward’ change. When the channel state governed by two control variables resides within the mutation region, the channel shifting progresses along path I as shown in Fig. 4a. If a transition from state A to C is required, the system initially bypasses C through a mutation, taking an upper branch. Then it returns to C to complete the state transformation, as illustrated in Fig. 4b. Conversely, if the state is outside the mutation region, the change occur along path III, directly transitioning from state A to C as shown in Fig. 4c. This latter process is continuous and single-valued, no abrupt transitions. An alternative pathway, denoted as path II, exists that mediates between these two conditions.

#### Determination of state variables and control variables for the sudden channel shifting of a wandering river

In this section, we incorporate cusp catastrophe theory to investigate the mechanisms underlying abrupt shifts in the channel dynamics of a wandering river.

##### The first control variable

The lateral stability coefficient, serving as a quantifiable representation of lateral channel stability, was chosen as the first control variable for this study. Within the context of riverbed evolution, Altunin's formula is employed, which represents the empirical relationship among channel bed width, bed slope, and flow discharge, providing a framework for investigating lateral stability in river systems:

1 $$ \varphi_{1} = \frac{B}{{B^{\prime}}} = 1 - \frac{{BJ^{0.2} }}{{Q^{0.5} }} $$

where $Q$ is the bed-forming discharge, $B$ is the river width corresponding to the bed-forming discharge, $B^{\prime}$ is the critical stable river width corresponding to the bed-forming discharge, and $J$ is the channel slope. The larger the value, the worse the lateral stability and the more unstable the riverbed.

While Altunin's formula can effectively capture the lateral stability of a riverbed, the scarcity of discharge data, particularly outside of hydrological stations, limits its applicability for computational analysis. Therefore, alternative methodologies are necessitated. Commonly, the width-to-depth ratio is utilized as an indicator of changes in cross-sectional shape. For the purposes of this study, fluctuations in this ratio were chosen to represent the channel's lateral stability. Generally, a larger variation in the width-to-depth ratio is indicative of increased instability in channel migration.

2 $$ \varphi_{1} = \frac{{\sqrt {B_{2} } }}{{H_{2} }} - \frac{{\sqrt {B_{1} } }}{{H_{1} }} $$

where $H_{1}$ and $H_{2}$ are the channel water depths before and after the riverbed change, $B_{1}$ and $B_{2}$ are the mean river widths corresponding to the bed-forming discharge before and after the riverbed change.

##### The second control variable

The longitudinal stability coefficient was identified as the second control variable in this study. Within the framework of riverbed evolution, Orlov postulated that a river's sediment transport capacity is fundamentally determined by longitudinal inertial forces, which are in turn influenced by the channel slope. Here, Orlov's formula provides a framework for investigating longitudinal stability in river systems:

3 $$ \varphi_{2} = \frac{{\gamma_{s} - \gamma }}{\gamma } \cdot \frac{{D_{50} }}{hJ} $$

where D_50_ is the median particle size of bed sand, $\gamma_{s}$ and $\gamma$ are the volume weights of sediment and water, respectively; *h* is the water depth, and *J* is the river slope corresponding to the bed-forming discharge.

Channel migration is intimately linked to intensive erosion and deposition of sediment within the riverbed. The extent of scouring and silting, which arises from imbalances in sediment transport, exerts a significant impact on riverbed evolution.

Considering a riverbed unit, the effect of scouring and silting is described by the following formula:

4 $$ I(t)\frac{{dF({\text{b}})}}{dx} = g({\text{b}}) - g({\text{s}}) $$

where $F(b)$ represents the boundary conditions described by mathematical equations, *t* represents the time, $I(t)$ stands for the riverbed inertia, $g({\text{b}})$ is the sediment transportation rate, which is a function of the shape and position of the boundary, and $g({\text{s}})$ represents the coming sediment rate of the riverbed unit. If the sediment transportation rate is higher than the coming sediment rate, $\frac{{dF({\text{b}})}}{dx}$ has a positive value, and the riverbed unit is scouring. If the sediment transportation rate is lower than the coming sediment rate, $\frac{{dF({\text{b}})}}{dx}$ is a negative value, and deposition happens. If $g({\text{b}}) = g({\text{s}})$, $\frac{{dF({\text{b}})}}{dx} = 0$, the deposition and erosion of the riverbed are balanced and stable.

The longitudinal stability of the riverbed can be measured by the change in erosion and silting strength. In this study, *f* was used to represent the erosion and silting change of the riverbed. If $f > 0$, the riverbed is silted; if $f < 0$, it is scoured. $f = 0$ indicates that the riverbed is not scoured or silted. The erosion and deposition of the riverbed increase with the |*f*| value, making the riverbed more unstable. Then, for consistency, the reciprocal of the erosion and silting index was set as the longitudinal stability index $\varphi_{2} = \frac{1}{f}$. The higher the φ_2_, the more stable the riverbed. When the riverbed is not scoured or silted, i.e., $\varphi_{2} \to \infty$, then the riverbed is the most stable.

The stability of channel shifting of a wandering river is principally determined by two factors: erosion and deposition of riverbed sediment, and alterations to the cross-sectional morphology of the riverbed. Consequently, the control variables for a model focused on sudden channel shifting should encompass these two essential components.

##### The state variable

Due to the comprehensive effect of water, sediment, riverbed, and boundary conditions, the channel state of a wandering river constantly changes. Here, $F = F(x,\;\mu ,\;\nu )$ was used to represent the channel change of the wandering LYR as a potential function. The state variable $x$ is a one-dimensional variable, and the control variables $(\mu ,\;\nu )$ are two-dimensional, with the first and second control variables.

The potential function expression of the cusp catastrophe model is as follows:

5 $$ V(x) = x^{4} + \mu x^{2} + \nu x $$

The state space is three-dimensional, like (*x*, *μ*, *ν*), $\mathrm{u}$ μ and ν are the control variables, and *x* is the state variable. The mutation manifold is obtained by the derivative of the potential function. The equation of the equilibrium hypersurface M, with wrinkles in a 3D space (*x*,* μ*, *ν*) is as follows:

6 $$ \frac{\partial V}{{\partial x}} = 4x^{3} + 2\mu x^{{}} + \nu $$

Based on the analysis of the mechanisms underlying the sudden channel shifting and its associated parameters, the cusp catastrophe model is considered suitable for investigating sudden channel shifts in wandering rivers.

In this model, the mainstream migration rate $l/t$ serves as the state variable. This rate is defined as the distance of mainstream migration per unit of time, which can be quantified by measuring the distance between two mainstream lines on the same cross-section within a specific time interval^51^. The mainstream migration rate refers to the distance of the mainstream migration per unit of time. The larger the $l/t$ value, the stronger the intensity of mainstream migration.

7 $$ \nu = \varphi_{2} ,\quad \mu = \varphi_{1} ,\quad x = l/t $$

In a stable channel state, the state variable has a unique extreme value, rendering the equilibrium positions on both the upper and lower leaves of the equilibrium surface stable. Conversely, in an unstable channel state, the state variable exhibits multiple extremal values, and the potential function in the middle of the equilibrium surface takes the maximum value.

#### Construction of the cusp catastrophe model for the sudden channel shifting of wandering river

Upon identifying the state and control variables, a model for the sudden channel shifting in wandering river was formulated. In this model, two control variables, denoted as φ_1_ and φ_2_, along with the state variable *x,* were incorporated into the standard potential function of the cusp catastrophe model. The potential function representing the sudden shifting of the wandering river can be described by the following model:

8 $$ F = l^{4} + \varphi_{2} l^{2} + \varphi_{1} l + R $$

It is a steady-state model. After a single derivation, Eq. (8) becomes as follows:

9 $$ F^{\prime}(l) = 4l^{3} + 2\varphi_{2} l + \varphi_{1} $$

**Figure 5:**
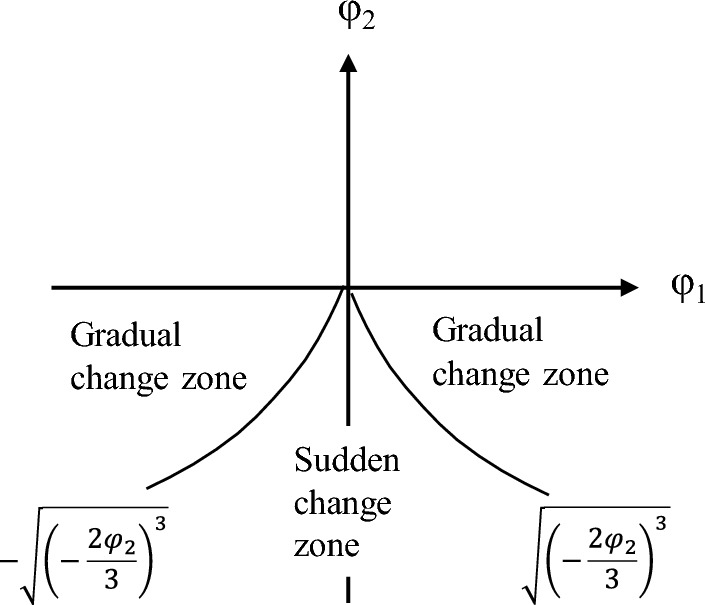
Equilibrium surface of the cusp catastrophe model.

The model is represented as a surface with a wrinkled part in a 3D space, φ_1_ is the regular control factor, and φ_2_ is the subdivision control factor. If φ_2_ > 0, its change only causes a smooth continuous change of the state variable *l*, and no extreme value appears. If φ_2_ < 0, the changes in state variables are no longer continuous, and wrinkles appear on the surface. At the edge of the fold are two extremum lines of the state variables. The folded area is a state jump area that is unstable (Fig. 5).

According to Cardano's discriminant method of the cubic equation of one variable, the bifurcation set equation is obtained:

10 $$ \Delta = 27\varphi_{{_{1} }}^{2} + 8\varphi_{{_{2} }}^{3} $$

Suppose Δ = 0; Eq. (9) has two real roots, and solving Eq. (10) leads to Eq. (11), which represents the critical state of the sudden channel shifting of wandering channels.

11 $$ \varphi_{1} = \pm \sqrt {\left( { - \frac{2}{3}\varphi_{2} } \right)^{3} } $$

If Δ > 0, Eq. (9) has one real root, and if Δ < 0, there are three real roots.

**Figure 6:**
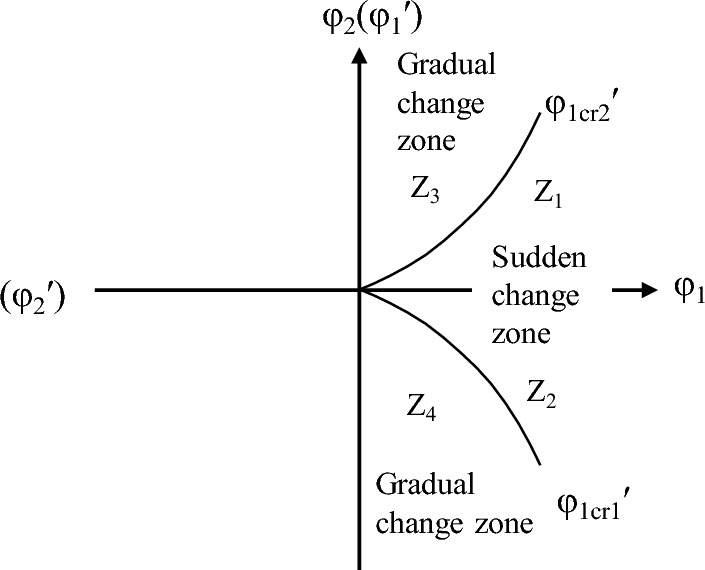
The cusp catastrophe model of sudden channel shifting after axes rotation.

The equilibrium surface is projected onto the plane defined of φ_1_ and φ_2_, as shown in Fig. 5. Upon projection, the folded surface transforms into a sharply angled area situated below the horizontal φ_1_ axis and the boundary line is symmetrical with respect to the longitudinal axis φ_2_. This suggests that φ_1_ attains a real value only when φ_2_ is negative. However, in the process of sudden channel shifting, the cross-sectional shape of the riverbed is always changing, and the riverbed may be scoured or silted. This indicates that the first control variable φ_1_ should be positive, and the second control variable φ_2_ can assume either positive or negative. Accordingly, we rotated the coordinate axes counterclockwise in Fig. 5, resulting in the configuration presented in Fig. 6. If erosion and deposition are fixed, the corresponding variation in the cross-sectional shape remains below the critical threshold for sudden channel shifting, as illustrated by Z_3_ and Z_4_ in Fig. 6, implying that the channel is undergoing a state of gradual change. Conversely, if the change in cross-sectional shape is fixed and the corresponding φ_2_ value falls below the critical threshold, the channel experiences a state of sudden change. It should be noted that both the riverbed's erosion–deposition dynamics and its cross-sectional shape are perpetually evolving.

To avoid sudden channel shifting, the control variables φ_1_ and φ_2_ should move counterclockwise to the recovery direction, meeting the criterion φ_1_ < ∣|φ_1cr_∣. To induce sudden channel shifting, the control variables should move clockwise in the recovery direction, meeting φ_1_ > ∣|φ_1cr_∣ and φ_2_ < ∣|φ_2cr_∣ criteria.

## Results of the sudden channel shifting for the wandering reach of the LYR

**Figure 7:**
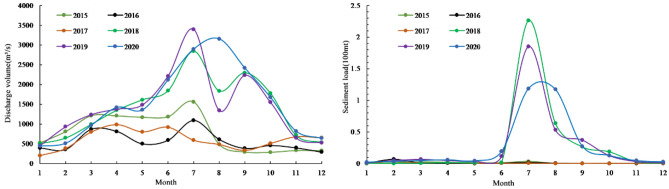
Monthly discharge volume and sediment load of the Huayuankou station from 2015 to 2020.

The wandering reach of the LYR, from Huayuankou to Gaocun, serves as the case study for this research (Fig. 2). Geometrical and topological data from 93 cross-sections were gathered between the years 2015 and 2019. The study period was subdivided into two intervals, 2015–2017 and 2018–2019, to account for variations in water and sediment conditions within the LYR. Specifically, the interval from 2015 to 2017 was characterized by reduced water inflow, as evidenced by average annual water volumes of about 209.5 × 10^8^ m^3^. Until 2018–2019, the average annual volume increased sharply, reaching 444.0 × 10^8^ m^3^ (Fig. 7). The period from May to September is considered the flood season, and from October to April next year is the non-flood season.

### Pattern of the sudden channel shifting for the wandering reach in 2015–2017

**Figure 8:**
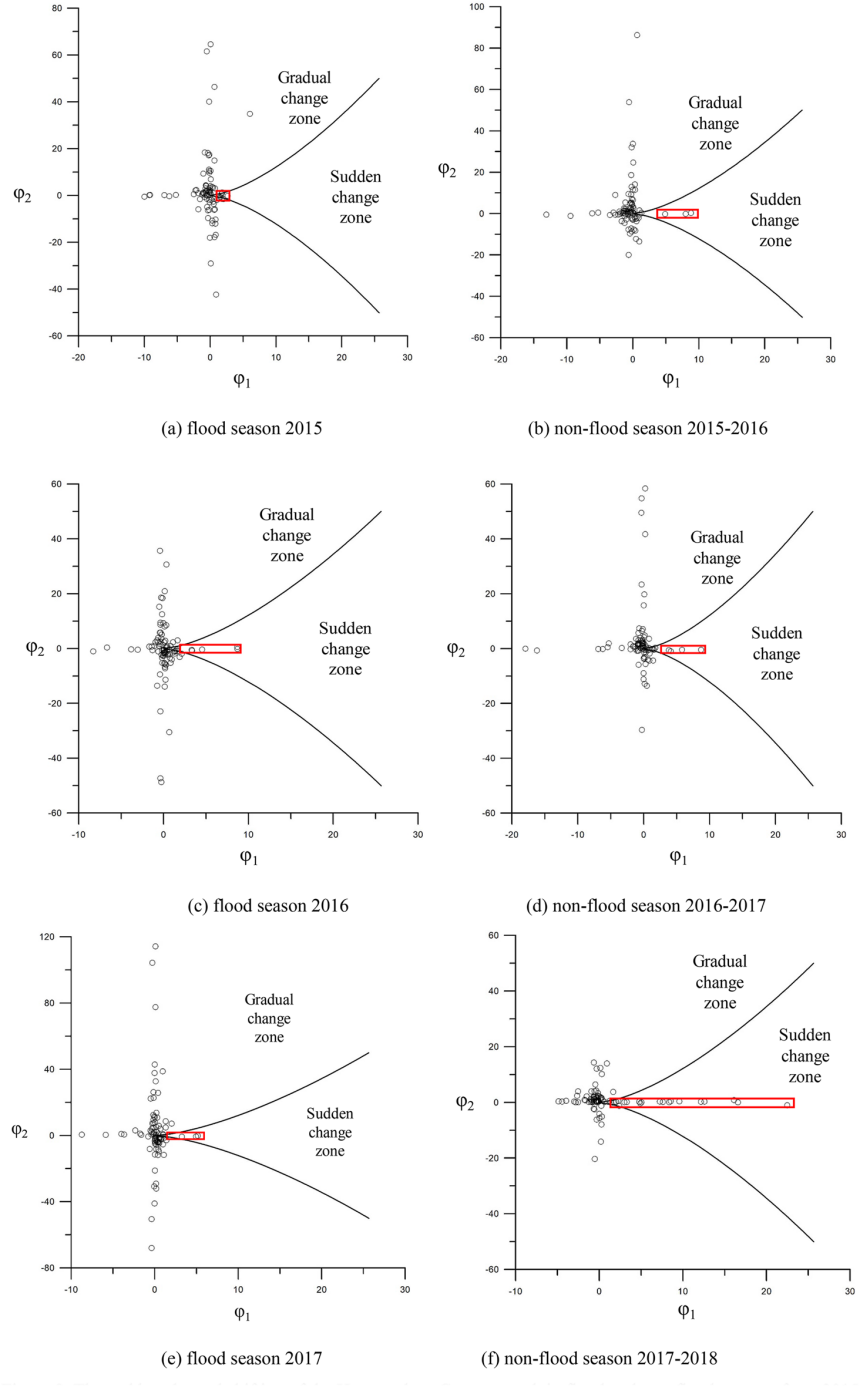
The sudden channel shifting of the Huayuankou-Gaocun reach in flood and non-flood seasons from 2015 to 2017 based on the cusp catastrophe model.

During the 2015 flood season, the Huayuankou-Gaocun reach exhibited overall satisfactory lateral and longitudinal stabilities, as shown in Fig. 8a. The lateral stability coefficient ranged from a maximum of 6.04 to a minimum of − 10.00, while the longitudinal stability coefficient varied between 64.56 and − 42.58. Notably, the Xiaodabin, Huihuizhai, and Heigangkou sections entered a critical state, indicative of imminent channel shifting, as detailed in Table 1. The majority of the remaining 93 sections demonstrated a propensity for gradual change.

**Table 1:** Change in sections' features in the sudden change zone during the 2015 flood season.

Section name	Before the 2015 flood season			After the 2015 flood season			Value of scouring and silting (10^6^ m^3^)
	Bankfull width (m)	$\sqrt{B/H}$	Slope	Bankfull width (m)	$\sqrt{B/H}$	Slope	
Xiaodabing	1180	9.52	− 0.0001	1165	11.33	0.0011	0.91
Huihuizhai	2719	11.80	− 0.0003	1115	14.46	0.0062	− 0.11
Heigangkou	718	3.77	0.0008	693	5.29	0.0206	− 0.56

In the non-flood season of 2015–2016, the Wuzhuang, Heishi, and Shiziyuan sections reached a critical state of sudden channel shifting, as illustrated in Fig. 8b and Table 2. The lateral stability coefficient fluctuated between 8.83 and − 13.15, while the longitudinal stability coefficient ranged from 86.27 to − 19.94.

**Table 2:** Change in sections' features in the sudden change zone during the 2015 non-flood season.

Section name	After the 2015 flood season			Before the 2016 flood season			Value of scouring and silting (10^6^ m^3^)
	Bankfull width (m)	$\sqrt{B/H}$	Slope	Bankfull width (m)	$\sqrt{B/H}$	Slope	
Wuzhuang	2407	21.06	0.0003	2858	13.05	− 0.0061	− 6.59
Heishi	3379	24.33	0.0002	3375	15.52	0.0225	3.75
Shiziyuan	1508	12.23	− 0.0009	1743	7.32	0.0059	− 5.41

For the 2016 flood season, the reach largely maintained good lateral and longitudinal stabilities, except for the Baochang, Xisanli, Huihuizhai, Zhuzhai, and Yulin sections, as shown in Fig. 8c and Table 3. In this season, lateral widening was more pronounced than in 2015. The lateral stability coefficient ranged from 8.70 to − 8.29, and the longitudinal stability coefficient varied between 35.66 and − 48.70.

**Table 3:** Change in sections' features in the sudden change zone during the 2016 flood season.

Section name	Before the 2016 flood season			After the 2016 flood season			Value of scouring and silting (10^6^ m^3^)
	Bankfull width (m)	$\sqrt{B/H}$	Slope	Bankfull width (m)	$\sqrt{B/H}$	Slope	
Baochang	1122	9.17	− 0.0006	958	12.52	− 0.0010	− 1.31
Xisanli	2113	7.80	− 0.0011	2710	16.51	0.0166	− 5.55
Huihuizhai	1010	9.21	0.0005	1010	12.52	0.0146	− 1.96
Zhuzhai	1403	11.80	− 0.0004	2661	20.49	− 0.0114	1.74
Yulin	1676	10.68	0	1649	15.20	− 0.0101	− 2.79

In the non-flood season of 2016–2017, lateral stability coefficients spanned from 8.71 to − 17.94, and longitudinal stability coefficients ranged from 54.82 to − 29.63. The Madu, Sanba, Sanguanmiao, and Wuqiu sections entered a critical state, as shown in Fig. 8d and Table 4.

**Table 4:** Change in sections' features in the sudden change zone during the 2016 non-flood season.

Section name	After the 2016 flood season			Before the 2017 flood season			Value of scouring and silting (10^6^ m^3^)
	Bankfull width (m)	$\sqrt{B/H}$	Slope	Bankfull width (m)	$\sqrt{B/H}$	Slope	
Madu	1986	10.15	− 0.0104	1953	15.97	0.0000	− 2.36
Sanba	444	3.54	− 0.0108	379	12.24	0.0008	− 2.35
Sanguanmiao	1554	12.79	0.0005	1372	16.61	0.0004	− 1.85
Wuqiu	2047	11.77	− 0.0121	2011	15.89	− 0.0022	− 1.04

During the 2017 flood season, the lateral stability coefficient ranged from 5.16 to − 8.74, and the longitudinal stability coefficient varied between 114.17 and − 67.95. Huayuankou, Baochang, Zhuzhai, and Yangzhuang sections transitioned into a critical state, as depicted in Fig. 8e and Table 5.

**Table 5:** Change in sections' features in the sudden change zone during the 2017 flood season.

Section name	Before the 2017 flood season			After the 2017 flood season			Value of scouring and silting (10^6^ m^3^)
	Bankfull width (m)	$\sqrt{B/H}$	Slope	Bankfull width (m)	$\sqrt{B/H}$	Slope	
Huayuankou	3057	17.01	–	3057	22.17	–	− 6.54
Baochang	958	12.80	0.0002	1052	14.83	0	0.14
Zhuzhai	740	4.28	0.0016	740	7.52	0	− 1.47
Yangzhuang	2495	14.00	− 0.0004	2495	18.93	− 0.0002	− 1.68

In the 2017–2018 non-flood season, the lateral stability coefficient fluctuated between 22.48 and − 4.87, while the longitudinal stability coefficient ranged from 14.35 to − 20.29. 15 sections, including Sunzhuang, Wuzhuang, Baochang, Maoan, Xinzhai, Yueshi, Guozhuang, Doumen, Weicheng, Heigangkou, Jinglonggong, Zhuzhai, Dongbaitou, Xinzhuang, and Zuozhaizha entered a critical state, as presented in Fig. 8f and Table 6. The number of sections in a critical state increased compared to the 2017 flood season and the 2016 non-flood season.

**Table 6:** Change in sections’ features in the sudden change zone during the 2017 non-flood season.

Section name	After flood the 2017 season			Before the 2018 flood season			Value of scouring and silting (10^6^ m^3^)
	Bankfull width (m)	$\sqrt{B/H}$	Slope	Bankfull width (m)	$\sqrt{B/H}$	Slope	
Sunzhuang	671	8.33	0.0009	2420	16.70	0	9.52
Wuzhuang	904	7.41	0	2418	15.02	0	5.58
Baochang	1052	14.83	0	2273	19.62	− 0.0006	4.37
Maoan	646	7.80	0.0008	1924	15.04	0.0006	3.31
Xinzhai	1132	12.12	− 0.0001	2010	20.67	0.0002	2.19
Yueshi	785	10.19	0.0002	1991	26.31	− 0.0001	1.22
Guozhuang	1892	21.54	0.0002	5098	26.58	− 0.0003	20.46
Doumen	601	7.17	0.0008	3545	23.76	0	18.58
Weicheng	2333	20.79	0	4324	23.80	0	10.49
Heigangkou	731	3.50	0.0004	1360	6.79	− 0.0010	5.38
Jinglonggong	649.8	5.39	− 0.0003	2189	17.56	− 0.0007	4.10
Zhuzhai	740	7.52	0	2661	20.11	− 0.0003	3.68
Dongbatou	673.7	7.16	− 0.0001	2475	29.64	− 0.0007	− 1.00
Xinzhuang	2542.2	14.98	− 0.0002	2132	19.89	0.0026	− 2.89
Zuozhaizha	717	5.93	− 0.0001	1765	15.51	− 0.0002	2.67

### Pattern of the sudden channel shifting for the wandering reach in 2018–2019

**Figure 9:**
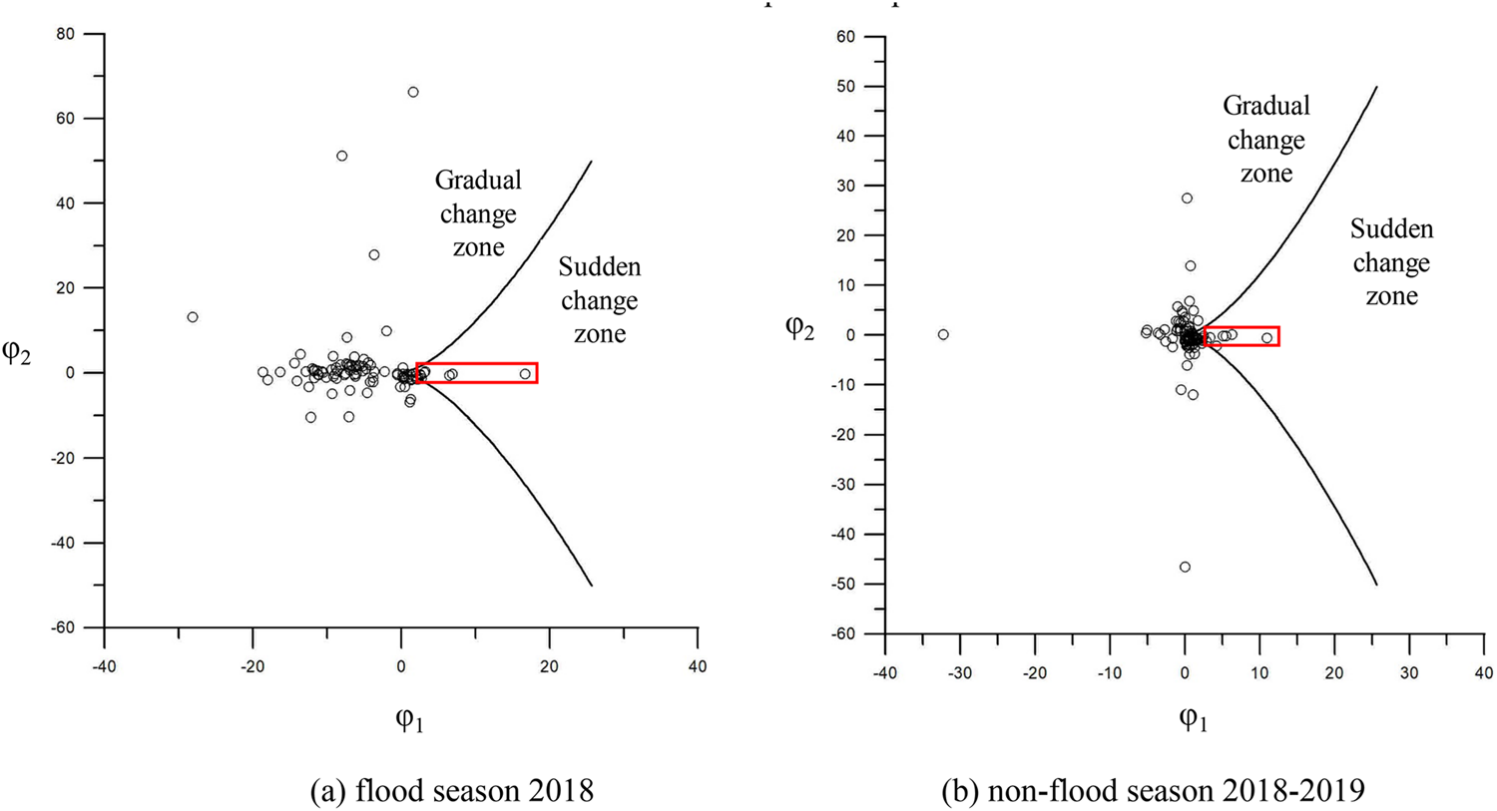
Channel sudden shifting of Huayuankou-Gaocun reach in flood season from 2018 to 2019 based on the cusp catastrophe model.

During the 2018 flood season, the Zhaolanzhuang, Huanglianji, Heishi, and Xuzhuang sections entered a critical state, indicative of imminent channel shifting, as illustrated in Fig. 9a. These sections exhibited pronounced lateral channel migration coupled with significant longitudinal scouring and silting, resulting in compromised channel stability. Compared to 2017, there was an increase in the $\sqrt{B/H}$ value, and the longitudinal slope of the river intensified, as detailed in Table 7. Overall, the Huayuankou-Gaocun reach demonstrated a tendency for gradual channel shifting.

**Table 7:** Change in sections' features in the sudden change zone during the 2018 flood season.

Section name	Before the 2018 flood season			After the 2018 flood season			Value of scouring and silting (10^6^ m^3^)
	Bankfull width (m)	$\sqrt{B/H}$	Slope	Bankfull width (m)	$\sqrt{B/H}$	Slope	
Zhaolanzhuang	1781	3.99	0.0049	1781	10.87	0.0033	− 3.85
Shiqiao	1496	7.99	0.0004	1496	10.51	0.0044	− 2.46
Huanglianji	3078	19.54	0.0004	3757	26.04	0.0054	− 1.69
Heishi	3384	27.51	0.0007	3387	44.23	0.0031	− 4.75
Xuzhuang	3859	18.98	0.0006	3859	22.17	0.0108	2.52

In the 2019 flood season, the Pochezhuang, Huanglianji, Yueshi, Heishi, Xuzhuang, and Chenqiao sections transitioned into a critical state, as shown in Fig. 9b and Table 8. These sections showed heightened susceptibility to sudden channel shifting.

**Table 8:** Change in sections' features in the sudden change zone during the 2019 flood season.

Section name	Before the 2019 flood season			After the 2019 flood season			Value of scouring and silting (10^6^ m^3^)
	Bankfull width (m)	$\sqrt{B/H}$	Slope	Bankfull width (m)	$\sqrt{B/H}$	Slope	
Pochezhuang	2332	11.26	0.0004	2332	14.61	0.0002	− 2.15
Huanglianji	2248	14.13	0.0005	3086	25.08	0.0003	− 1.62
Yueshi	1991	28.33	0.0000	1991	32.52	0.0000	− 0.45
Heishi	3327	38.46	0.0003	3402	44.73	0.0001	7.31
Xuzhuang	3859	20.26	0.0000	3856	25.31	0.000	− 5.19
Chenqiao	1653	8.52	0.0009	1653	14.01	0.0009	− 5.13

**Figure 10:**
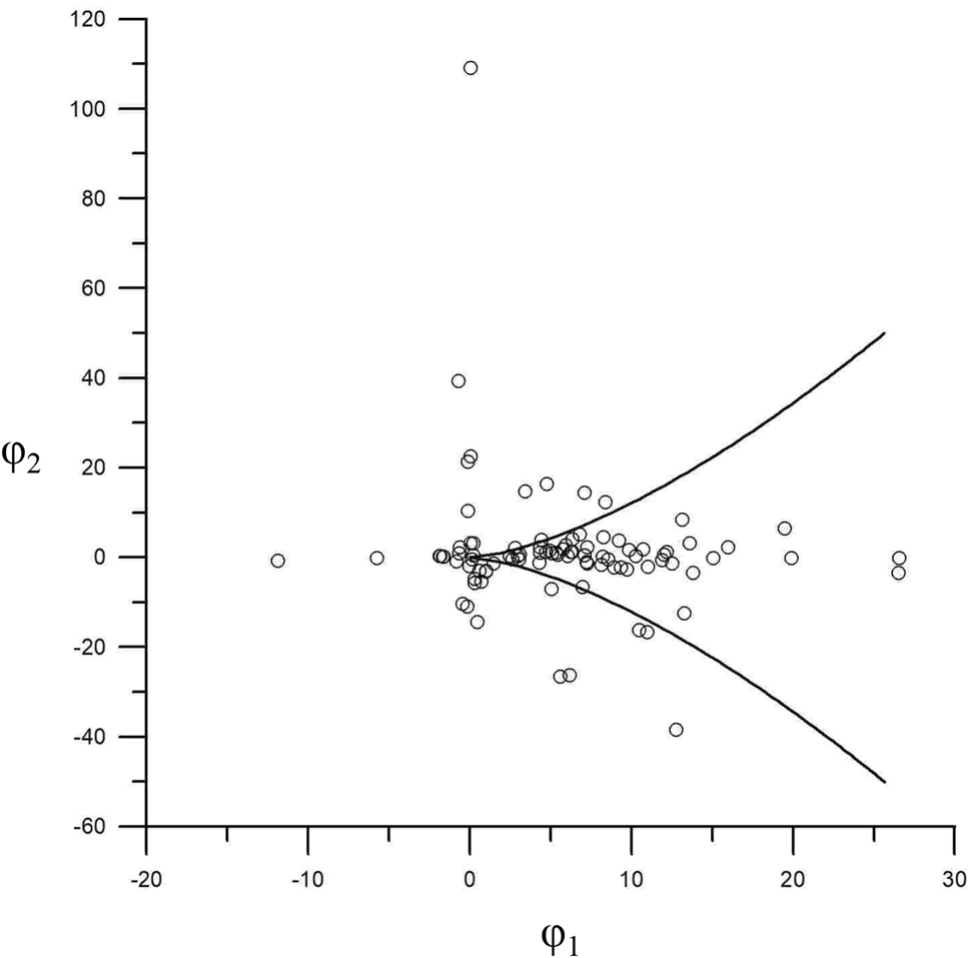
The sudden channel shifting of the Huayuankou-Gaocun reach in the 2018–2019 non-flood season based on the cusp catastrophe model.

During the non-flood season of 2018–2019, a distinct shift in channel behavior was observed, as depicted in Fig. 10. Approximately half of the 93 surveyed sections in the reach downstream of Jinglonggong entered a critical state, deviating from previous observations. This indicates an elevated risk of sudden channel shifting in these sections.

## Conclusions and discussion

### Conclusions

In this study, we conducted a comprehensive assessment of the channel stability of the LYR's wandering reach at the section scale for the period 2015–2019, following the construction of the Xiaolangdi Dam. Our findings indicate that the stability of this reach is primarily governed by two key factors: longitudinal erosion–deposition patterns and cross-sectional morphological variations. We have established a novel analytical model grounded in cusp catastrophe theory to quantitatively assess such channel shifts. Through an analysis of the LYR's wandering reach, we observed that both lateral and longitudinal stabilities in the Huayuankou-Gaocun section were generally favorable, with most selected sections demonstrating a propensity for gradual change. However, notable exceptions were found in the Xiaodabin-Jinglonggong and Huayuankou-Zhaolanzhuang reaches, which tended to experience sudden channel shifting during the period from 2015 to 2017, excluding the 2018–2019 non-flood season. In the latter non-flood period, nearly half of the sections in the downstream reach from Jinglonggong displayed instability. Our findings closely align with empirical observations from the Lower Yellow River.

### Discussion

To assess the proclivity for sudden channel shifting in the wandering reach of the LYR between 2015 and 2019, we utilized satellite imagery and systematic measurements of mainstream and bank lines. Our analysis indicated a general stability across the entire study reach during this period, with notable exceptions in the Xiaodabin-Jinglonggong and Huayuankou-Zhaolanzhuang reaches, which displayed instability.

The channel adjacent to the Huayuankou-Zhaolanzhuang section exhibited a progressive widening, with a prominent sandbar emerging mid-river^7^. Multiple channels were noted, and the mainstream migrated in excess of 2000 m over the study period. These observations corroborate earlier findings related to channel instability during specific flood seasons in different sections, including the Huayuankou section in 2017, the Zhaolanzhuang section in 2018, and the Pochezhuang section in 2019.

**Figure 11:**
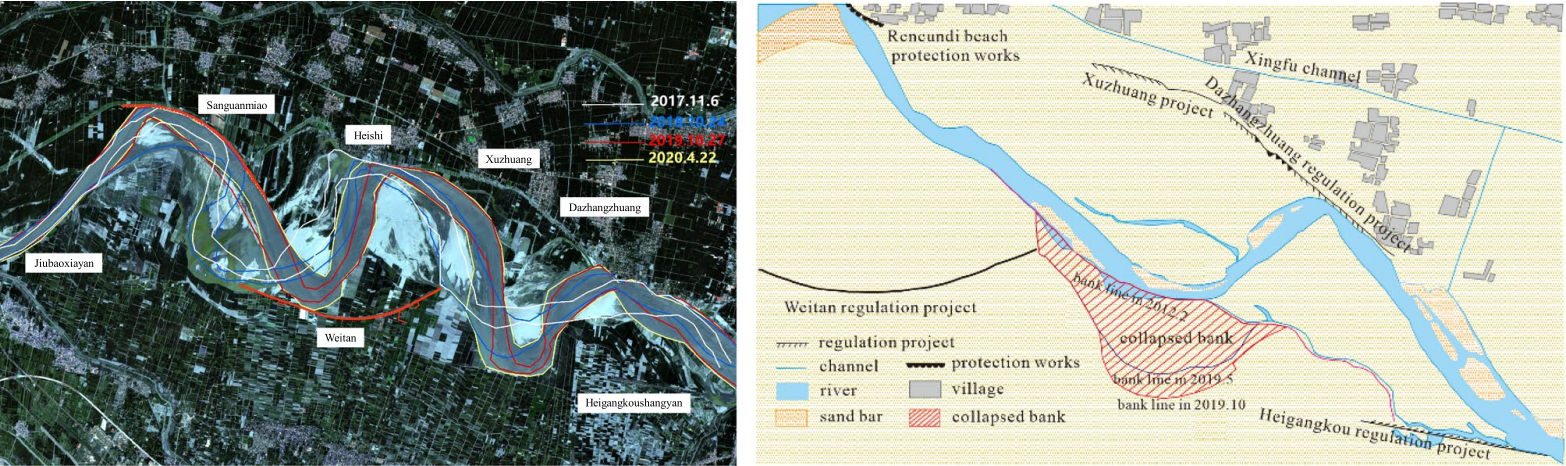
Channel shifting near the Sanguanmiao-Weitan reach of the lower Yellow River in 2017–2020. The satellite imagery was obtained from https://www.usgs.gov/ and the map was prepared by the co-author with the help of ArcGIS 10.8 (http://www.esri.com/software/arcgis) and does not require any permission from anywhere.

An anomalous river pattern was first identified in the Sanguanmiao-Weitan reaches in 2012. Initially, the river displayed a slow rate of change with a bending coefficient of approximately 2.3. However, by 2017, a marked increase in bank collapse and sudden channel shifting was observed, with the bending coefficient rising to 2.4 by 2018. Between May and October 2019, the mainstream shifted 380 m southward, and the collapsed bank line extended to 3.6 km, covering an area of about 84 hectares (Fig. 11). These observations align with previous research on various river sections across different seasons, thereby validating the model proposed in this paper as a reliable method for monitoring and analyzing sudden channel shifting.

**Figure 12:**
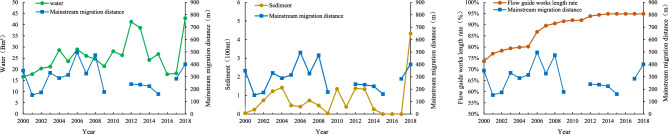
Average mainstream migration distance and water and sediment conditions of wandering channel in the lower Yellow River in 2000–2018 (ratio of flow guide works length means ratio of total length of flow guide works on both sides to the total length of the river section).

Hydrological and sedimentary factors significantly influence river path stability, particularly in inherently unstable wandering river systems. After 2014, reduced discharge and sediment deposition led to progressive erosion, contributing to sharp river bends in the Sanguanmiao-Weitan reaches (Fig. 12)^7^. Ineffective flow guide works are the second factor contributing to the formation of sharp bends^52^. As a result, an abnormal river pattern occurred in this section with a continuous process of low discharge and few sediments into the LYR. Beginning in 2018, an increase in water and sediment flux into the LYR (Figs. 7 and 12) accelerated river evolution, culminating in sudden channel shifting occurring in the unstable Xiaodabin-Jinglonggong reaches^7^. Additionally, high flood events played an important role in flow course migration and exacerbated the likelihood of sudden channel shifting in the study reach. Cutoff events near the Gaocun section and before Huayuankou occurred during the 2020 high flood when the maximum discharges at the Huayuankou hydrometric station reached 5180 m^3^/s, the highest value since the operation of the Xiaolangdi Reservoir^7,47^.

The phenomenon of sudden channel shifting in wandering rivers presents significant challenges for water supply and flood control management. While the cusp catastrophic model performed well in analyzing the LYR's channel stability, its broader applicability to other wandering rivers remains to be investigated. Moreover, this study primarily focuses on natural factors affecting channel shifting, without accounting for anthropogenic impacts. The lack of consideration for anthropogenic impacts is a limitation of this study. Future research should explore the role of human activity in channel behavior and assess the potential impact artificial works on mitigating or exacerbating channel instability. Such inquiries would offer valuable insights into predicting future occurrences of sudden channel shifting in wandering rivers.

## Data Availability

The data presented in this study are available on request from the corresponding authors.
